# Supplementary material for: Aerobic mechanochemical reversible-deactivation radical polymerization
Source: Nat Commun. 2024 Jul 22;15:6179. doi: 10.1038/s41467-024-50562-z (PMC11263483; doi:10.1038/s41467-024-50562-z)
Supplement: Supplementary file 1 — Supplementary Information [file 41467_2024_50562_MOESM1_ESM.pdf]

Supplementary Information for

Aerobic Mechanochemical Reversible-Deactivation Radical Polymerization

Haoyang Feng[1], Zhe Chen[2], Lei Li[1], Xiaoyang Shao[1], Wenru Fan[1], Chen Wang[1], Lin Song[1], Krzysztof Matyjaszewski*[3], Xiangcheng Pan*[2] & Zhenhua Wang*[1]

[1]Frontiers Science Center for Flexible Electronics (FSCFE) & Institute of Flexible Electronics (IFE), Northwestern Polytechnical University, Xi'an 710072, China

[2]State Key Laboratory of Molecular Engineering of Polymers, Department of Macromolecular Science, Fudan University, Shanghai 200438, China

[3]Department of Chemistry, Carnegie Mellon University, 4400 Fifth Avenue, Pittsburgh,10 PA 15213, USA

Corresponding author

Correspondence to: matyjaszewski@cmu.edu, panxc@fudan.edu.cn, iamzhwang@nwpu.edu.cn

Supplementary Information includes:

Supplementary Fig. 1 ^{1}H NMR spectroscopy in $CDCl_3$ of the pristine polymerization
Supplementary Fig. 2 ^{1}H NMR spectra in $CDCl_3$ of controls
Supplementary Fig. 3 ^{1}H NMR spectroscopy in $CDCl_3$ of polymerization under 30 °C heating
Supplementary Fig. 4 GPC traces evolution of polymerization under 30 °C heating and the pristine polymerization
Supplementary Fig. 5 GPC traces evolution of entry 30 Hz at timed intervals
Supplementary Fig. 6 ^{1}H NMR spectra in $CDCl_3$ of entry 30 Hz at timed intervals
Supplementary Fig. 7 GPC traces evolution of entry 10 Hz at timed intervals
Supplementary Fig. 8 ^{1}H NMR spectra in $CDCl_3$ of entry 10 Hz at timed intervals
Supplementary Fig. 9 GPC traces evolution of entry 20 Hz at timed intervals
Supplementary Fig. 10 ^{1}H NMR spectra in $CDCl_3$ of entry 20 Hz at timed intervals
Supplementary Fig. 11 ^{1}H NMR spectra in $CDCl_3$ of aerobic mechano-RAFT with different DP_T
Supplementary Fig. 12 GPC traces evolution of aerobic mechano-RAFT with DP_T = 1000
Supplementary Fig. 13 MALDI-TOF mass spectroscopy of P*n*BA
Supplementary Fig. 14 GPC trace of P*n*BA
Supplementary Fig. 15 MALDI-TOF mass spectroscopy of P*n*BA in enlarge view
Supplementary Fig. 16 Chain extension polymerization reaction formula

Supplementary Fig. 17 FTIR spectra of Et_3B and Et_3B-PyOMe complex before and after oxidation
Supplementary Fig. 18 ^1H NMR spectra in $CDCl_3$ of different monomers after Process 1
Supplementary Fig. 19 ^1H NMR spectroscopy in $CDCl_3$ of MMA monomer after Process 1
Supplementary Fig. 20 GPC traces evolution of different monomers after Process 1
Supplementary Fig. 21 ^1H NMR spectroscopy in $CDCl_3$ of MMA monomer after Process 2
Supplementary Fig. 22 GPC trace of MMA monomer after Process 2
Supplementary Fig. 23 ^1H NMR spectroscopy in $CDCl_3$ of styrene monomer after Process 2
Supplementary Fig. 24 ^1H NMR spectroscopy in $CDCl_3$ of vinyl naphthalene monomer after Process 2
Supplementary Fig. 25 ^1H NMR spectroscopy in $CDCl_3$ of vinyl carbazole monomer after Process 2
Supplementary Fig. 26 GPC traces of styrene, vinyl naphthalene and vinyl carbazole after Process 2
Supplementary Fig. 27 ^1H NMR spectra in DMSO-d6 of NIPAM monomer after Process 3
Supplementary Fig. 28 ^1H NMR spectroscopy in DMSO-d6 of NPA monomer after Process 3
Supplementary Fig. 29 GPC trace (in DMF solution) of NPA monomer after Process 3
Supplementary Fig. 30 GPC traces (in DMF solution) of NIPAM with different DP_T after Process 3
Supplementary Fig. 31 ^1H NMR spectroscopy in $CDCl_3$ of the copolymerization of *n*BA and NIPAM based on aerobic mechano-RAFT
Supplementary Fig. 32 GPC trace of the copolymerization of *n*BA and NIPAM based on aerobic mechano-RAFT
Supplementary Fig. 33 ^{13}C NMR spectroscopy (DEPT135, CH_3/CH positive and CH_2 negative, in $CDCl_3$) of the copolymerization of *n*BA and NIPAM based on aerobic mechano-RAFT

Supplementary Table 1 Aerobic mechano-RAFT with different initiator dosages

Supplementary Table 2 Aerobic mechano-RAFT with different LAG
Supplementary Table 3 Aerobic mechano-RAFT with different CTAs

Supplementary Table 4 Aerobic mechano-RAFT with different milling balls

Supplementary Table 5 Aerobic mechano-RAFT with different temperature

Supplementary Table 6 Aerobic mechano-RAFT with different air volume

Synthesis methods

Supplementary references

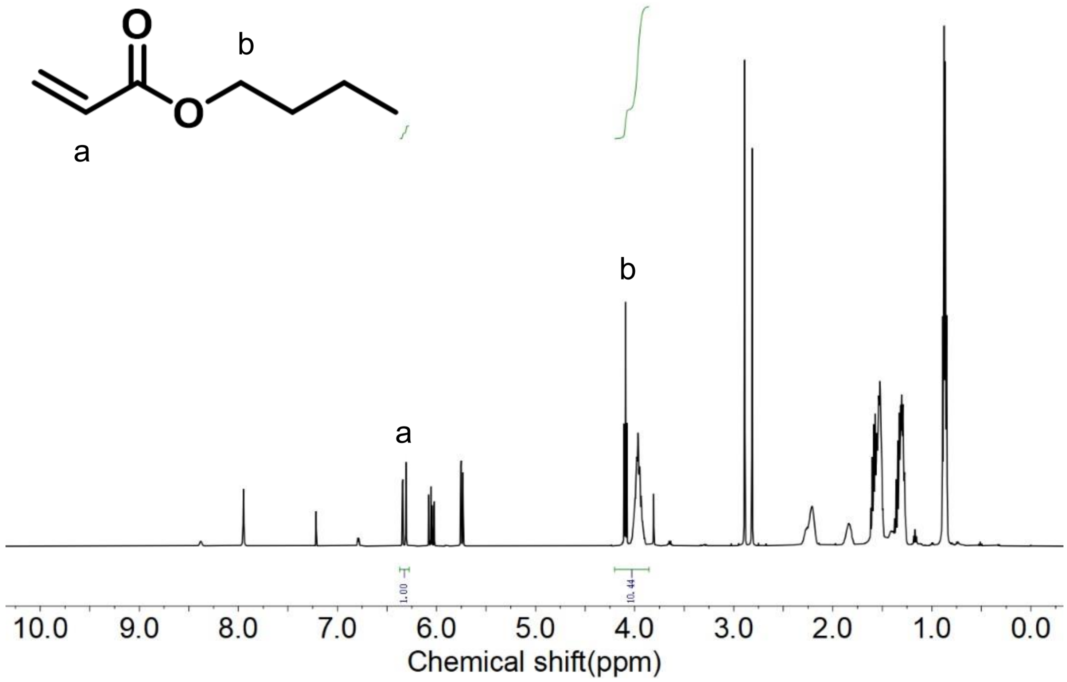

Supplementary Fig. 1 ^{1}H NMR spectroscopy in $CDCl_3$ of the pristine polymerization

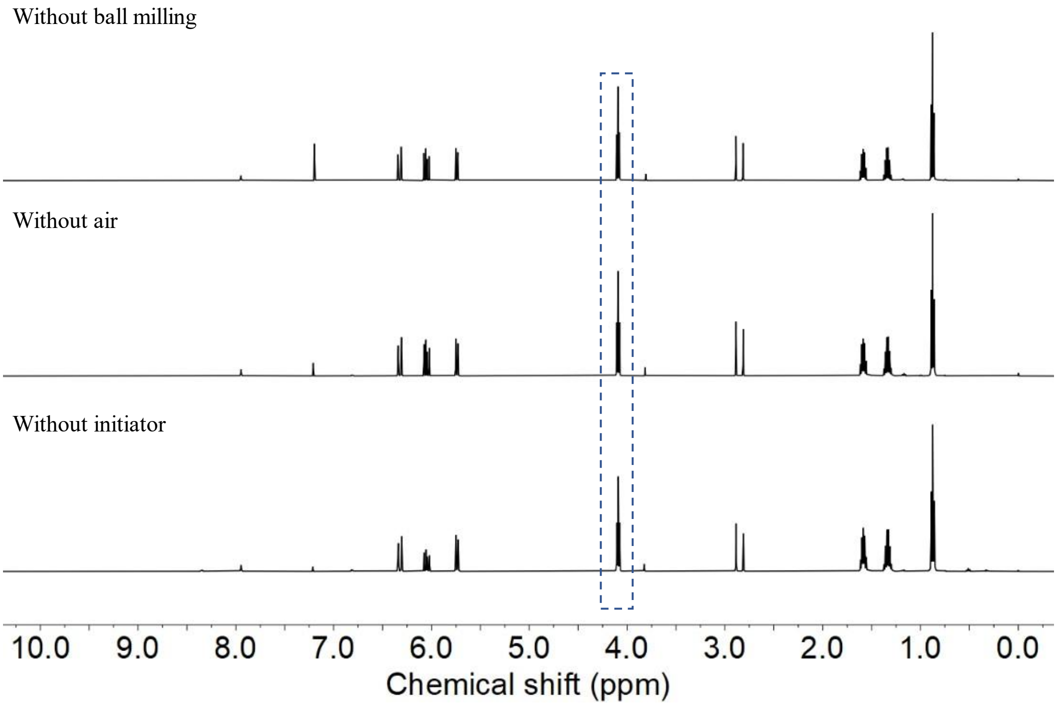

Supplementary Fig. 2 ^{1}H NMR spectra in $CDCl_3$ of controls

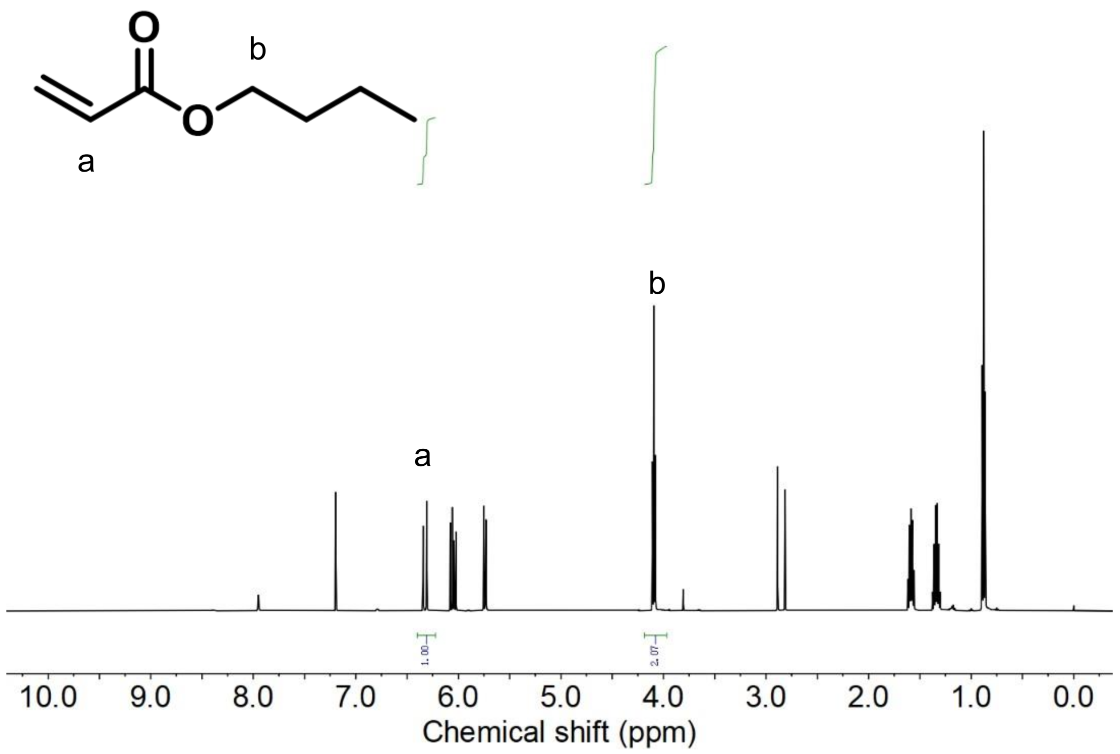

Supplementary Fig. 3 ^{1}H NMR spectroscopy in $CDCl_3$ of polymerization under 30 °C heating

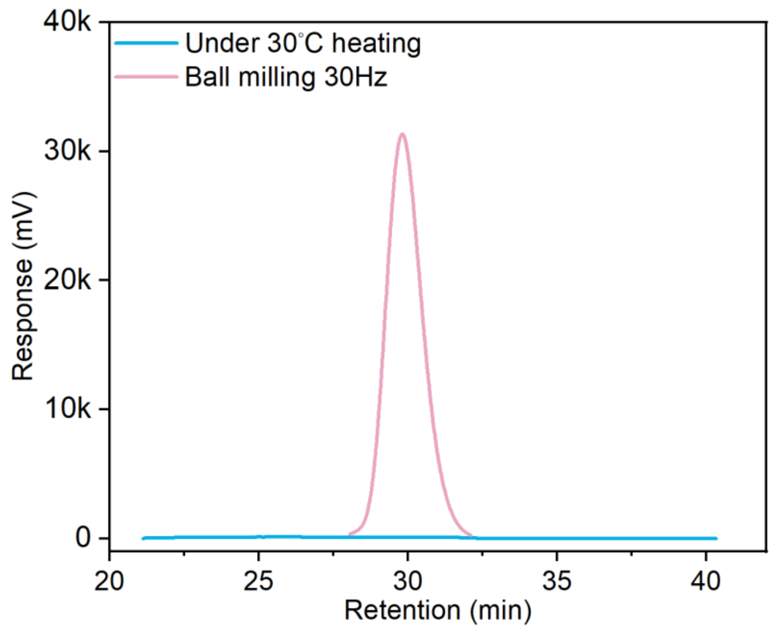

Supplementary Fig. 4 GPC traces evolution of polymerization under 30 °C heating and the pristine polymerization

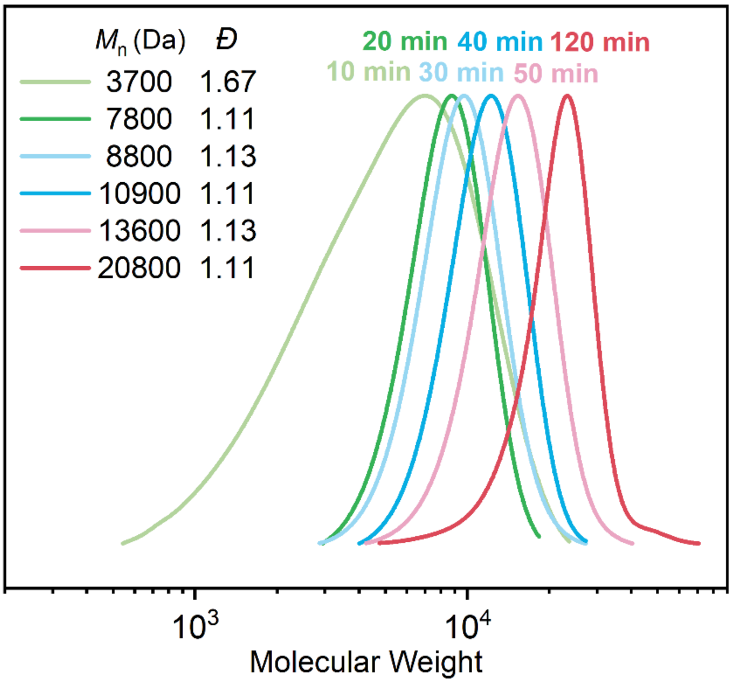

Supplementary Fig. 5 GPC traces evolution of entry 30 Hz at timed intervals

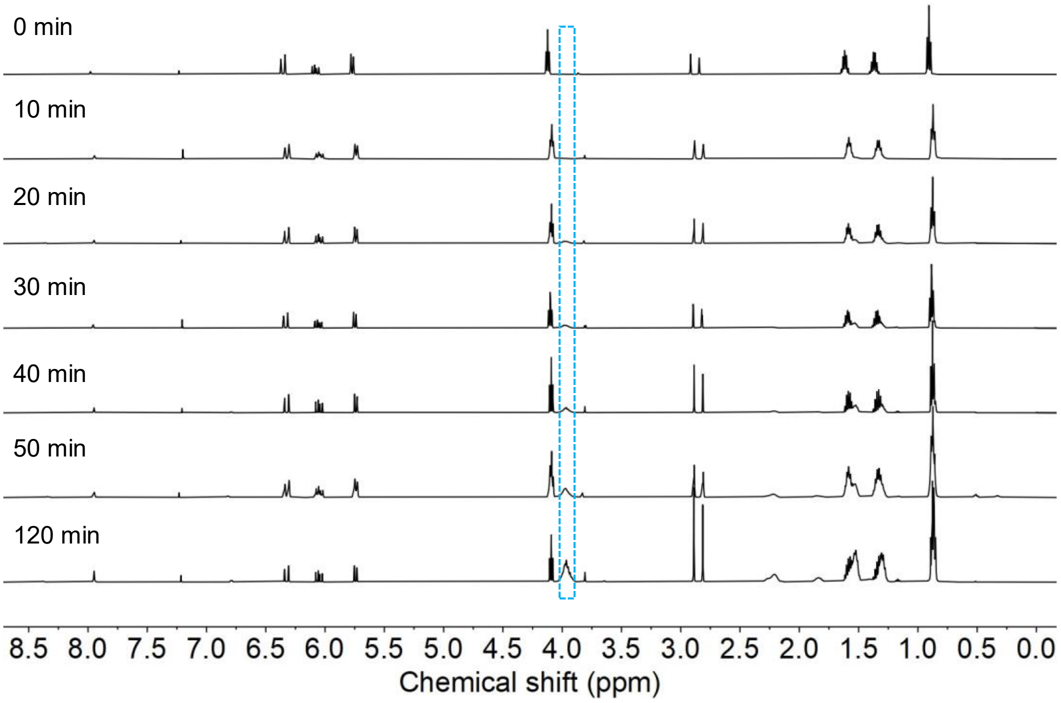

Supplementary Fig. 6 ^{1}H NMR spectra in $CDCl_3$ of entry 30 Hz at timed intervals

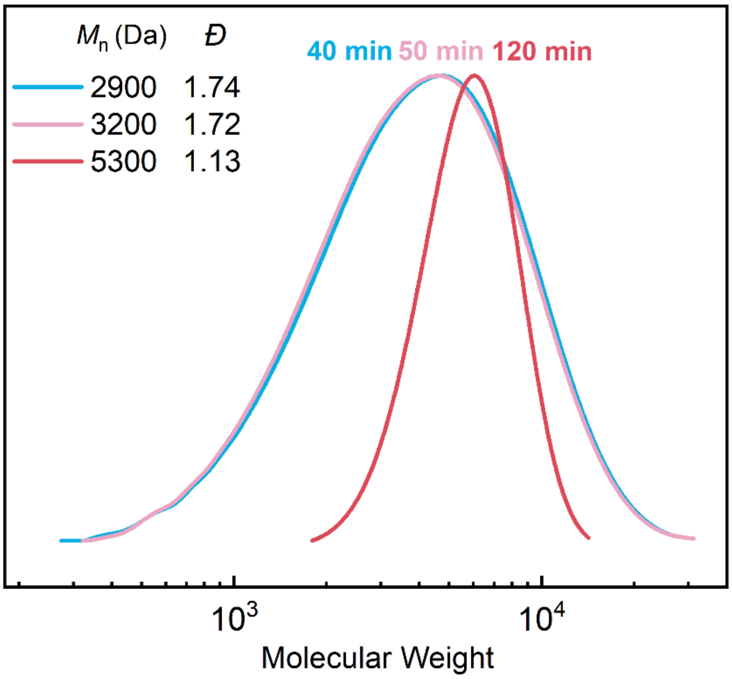

Supplementary Fig. 7 GPC traces evolution of entry 10 Hz at timed intervals

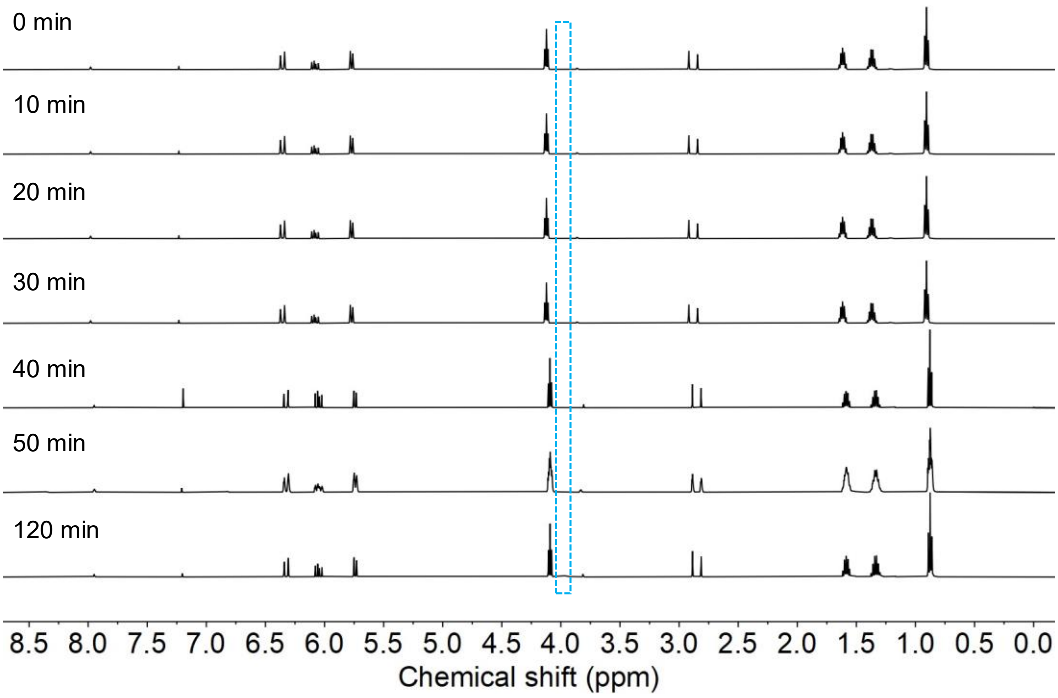

Supplementary Fig. 8 ^{1}H NMR spectra in $CDCl_3$ of entry 10 Hz at timed intervals

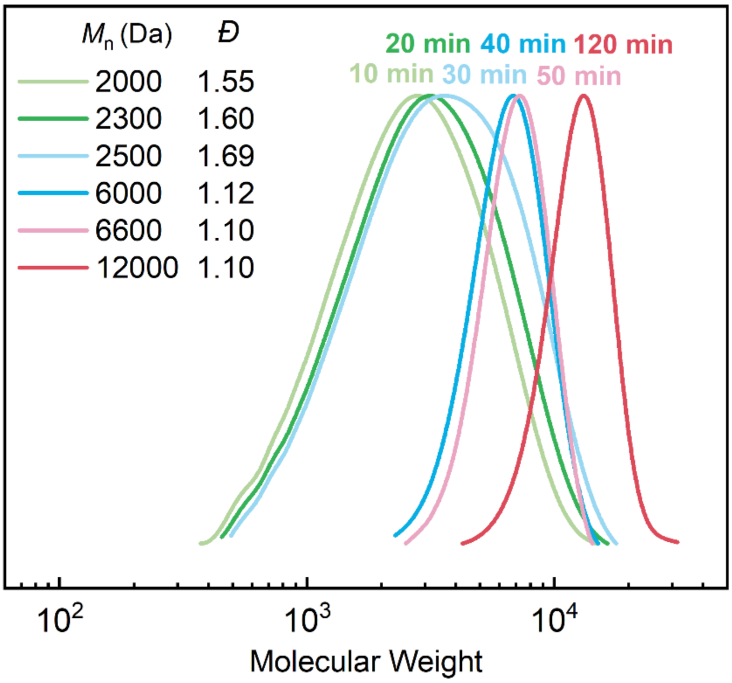

Supplementary Fig. 9 GPC traces evolution of entry 20 Hz at timed intervals

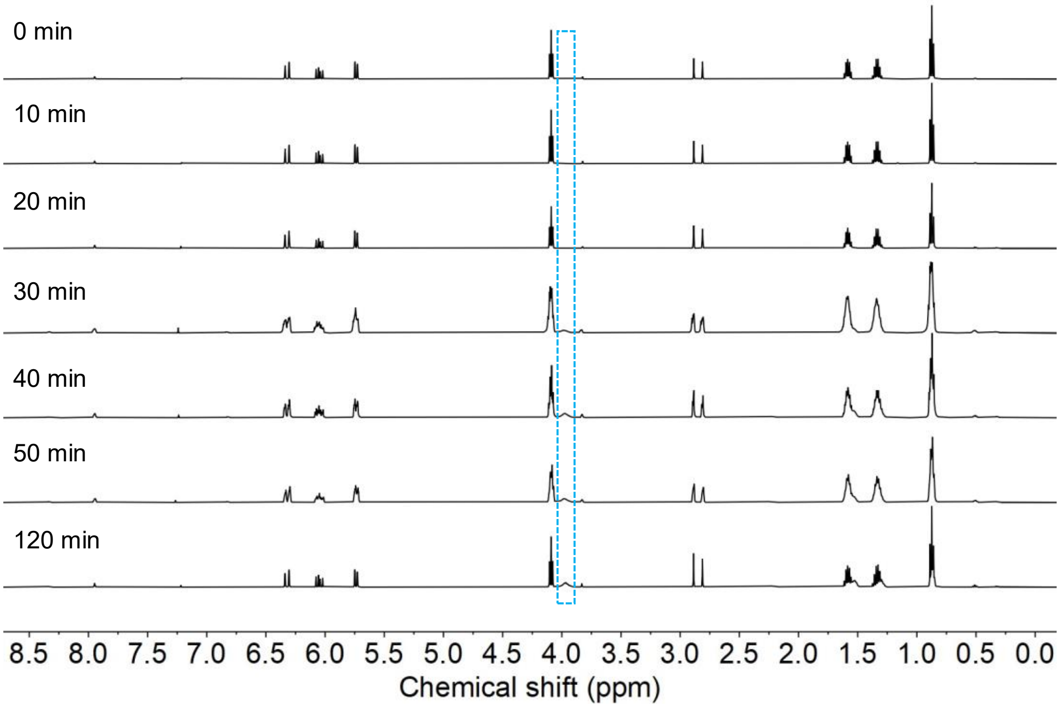

Supplementary Fig. 10 ^{1}H NMR spectra in $CDCl_3$ of entry 20 Hz at timed intervals

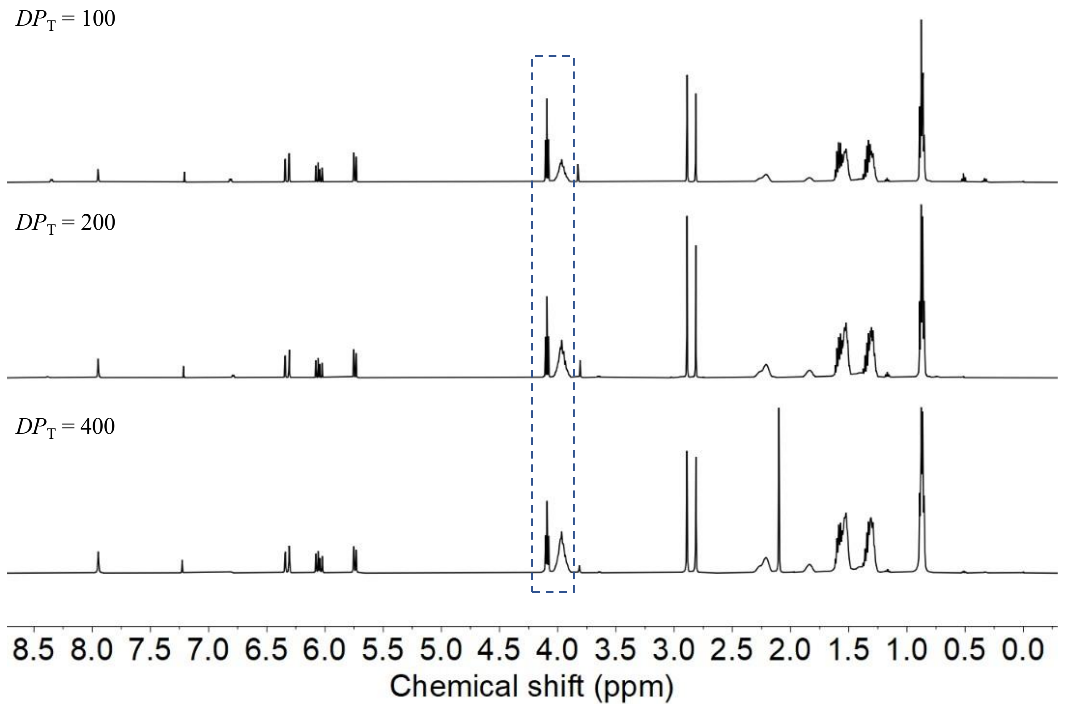

Supplementary Fig. 11 ^{1}H NMR spectra in $CDCl_3$ of aerobic mechano-RAFT with different DP_T

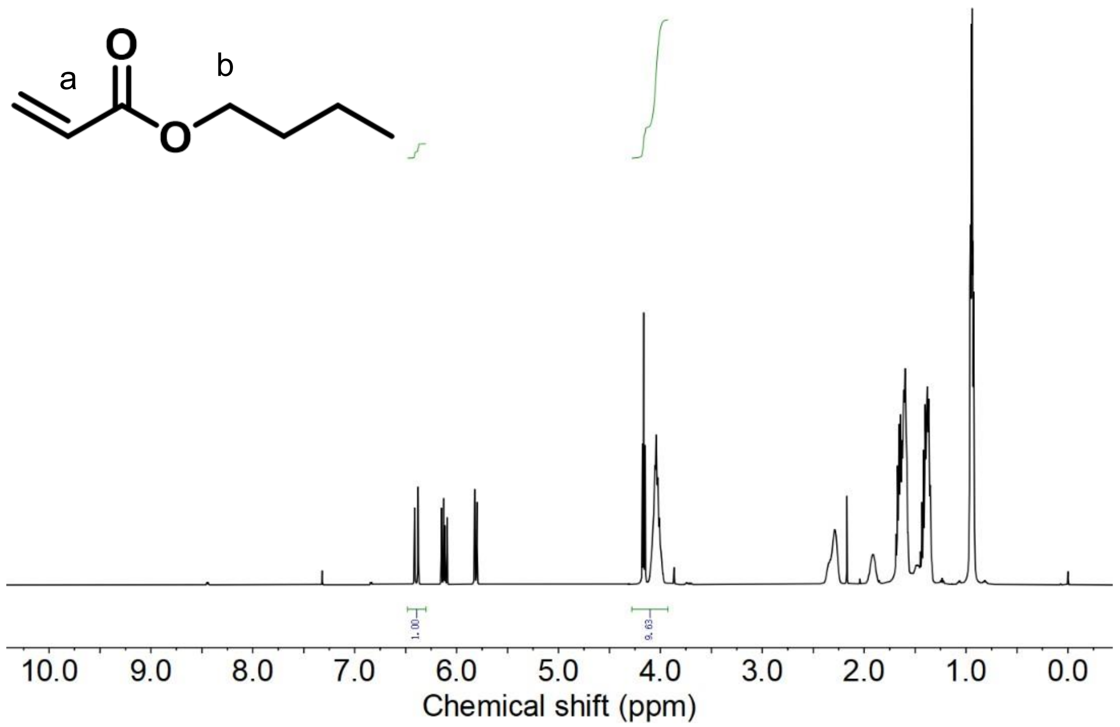

Supplementary Fig. 12 ^{1}H NMR spectroscopy in $CDCl_3$ of aerobic mechano-RAFT with DP_T =1000

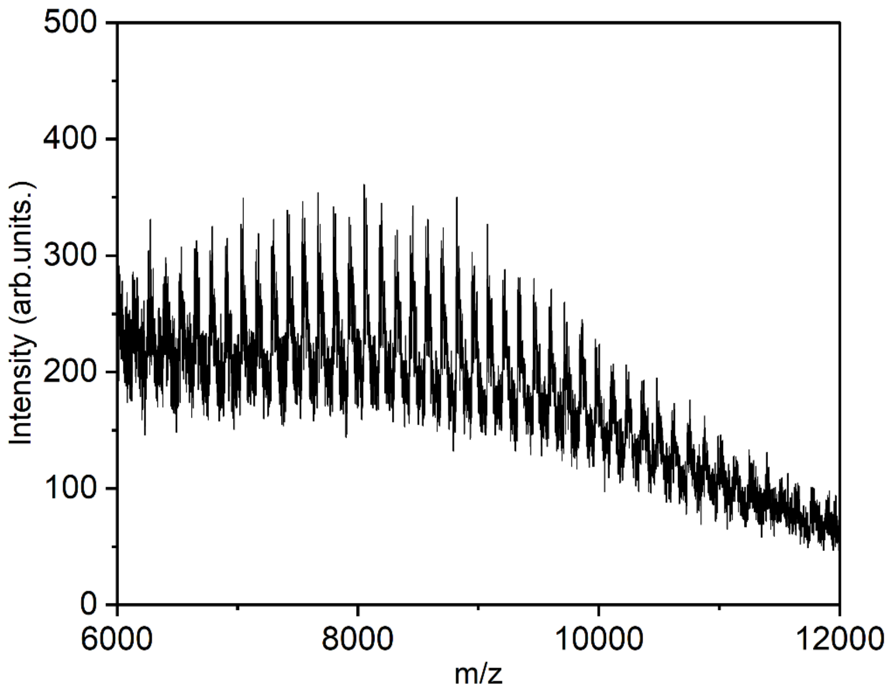

Supplementary Fig. 13 MALDI-TOF mass spectroscopy of P*n*BA

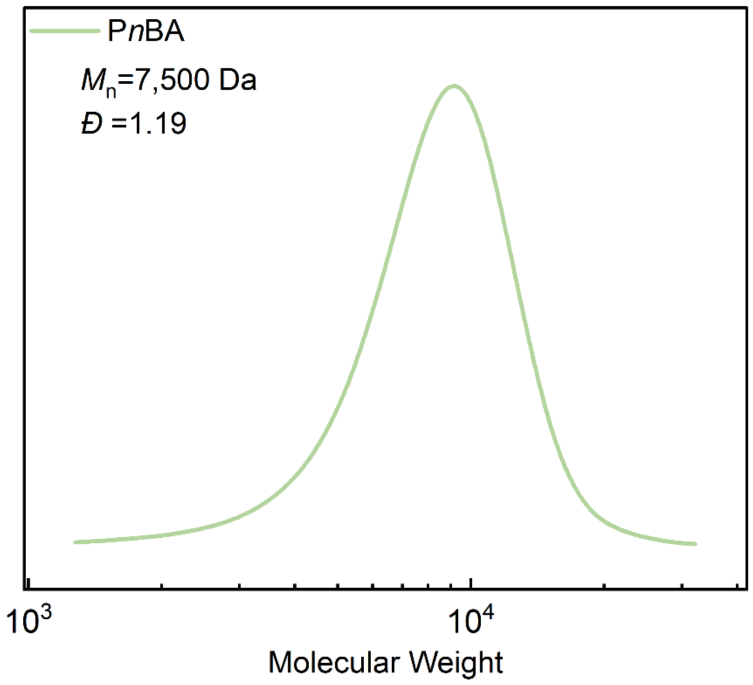

Supplementary Fig. 14 GPC trace of P*n*BA

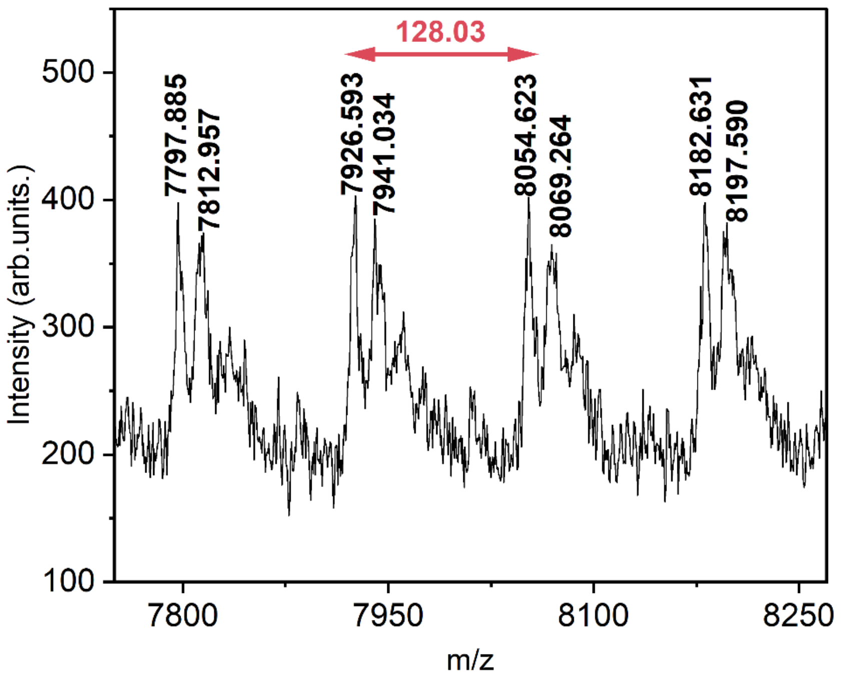

Supplementary Fig. 15 MALDI-TOF mass spectroscopy of P*n*BA in enlarge view

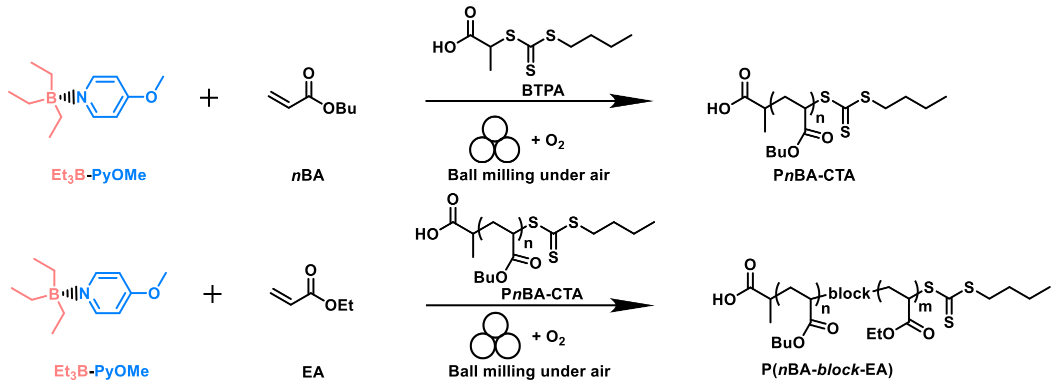

Supplementary Fig. 16 Chain extension polymerization reaction formula

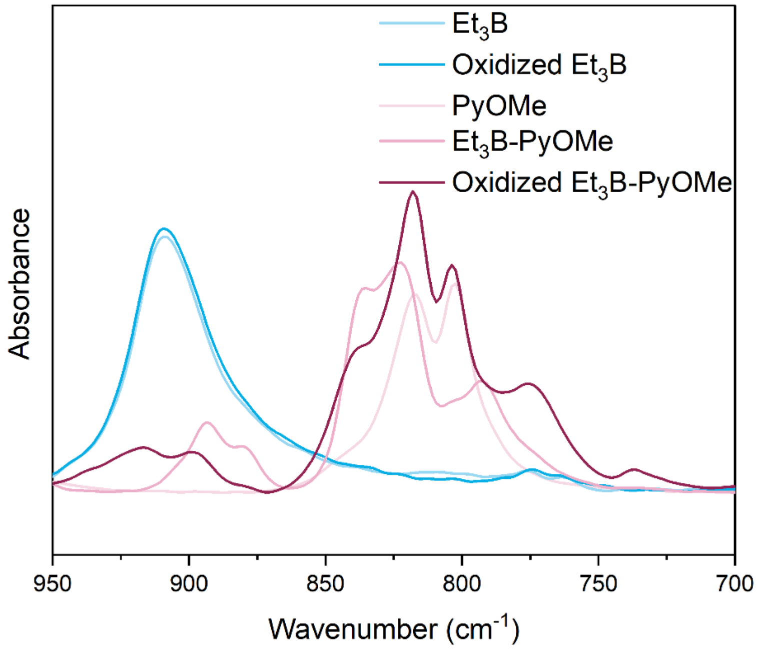

Supplementary Fig. 17 FTIR spectra of Et_3B and Et_3B-PyOMe before and after oxidation

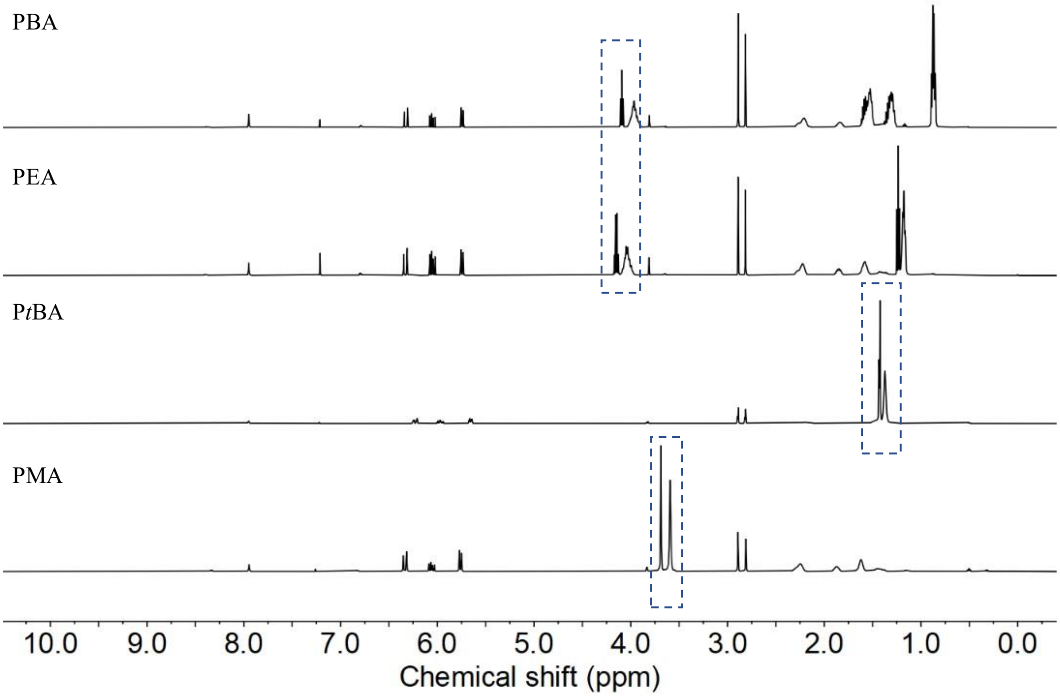

Supplementary Fig. 18 ^{1}H NMR spectra in $CDCl_3$ of different monomers after Process 1

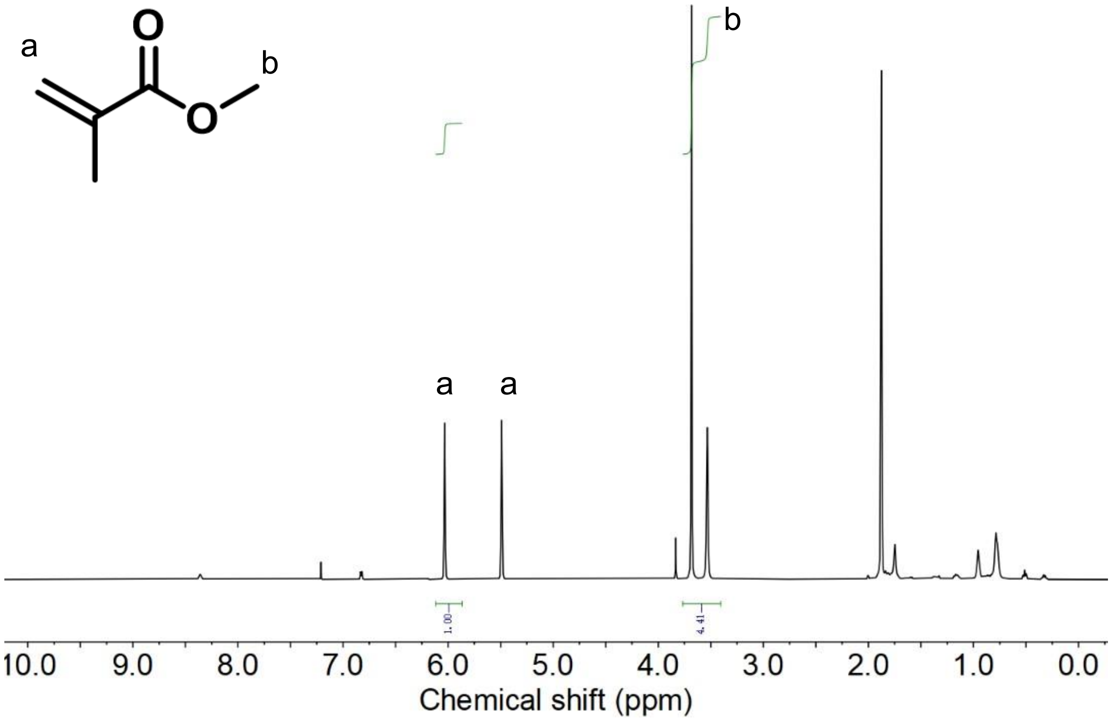

Supplementary Fig. 19 ^{1}H NMR spectroscopy in $CDCl_3$ of MMA monomer after Process 1

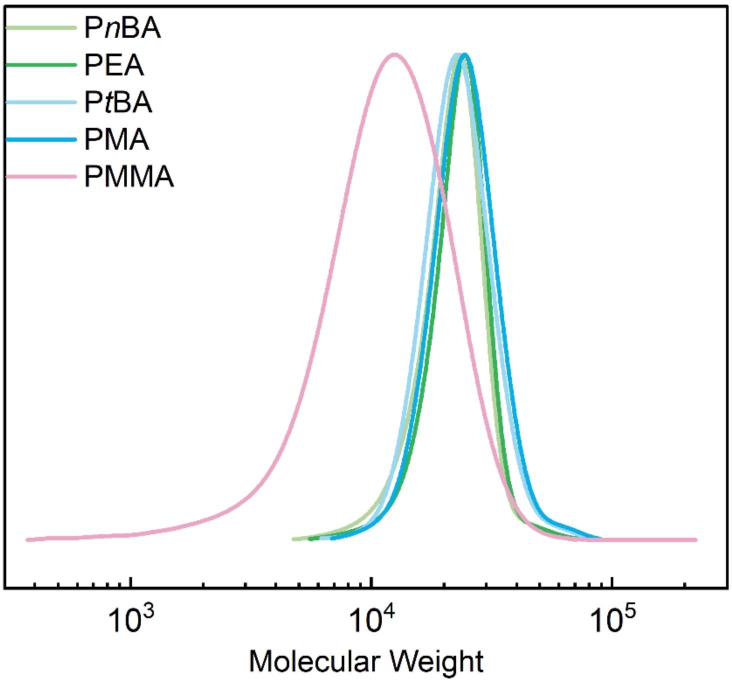

Supplementary Fig. 20 GPC traces of different monomers after Process 1

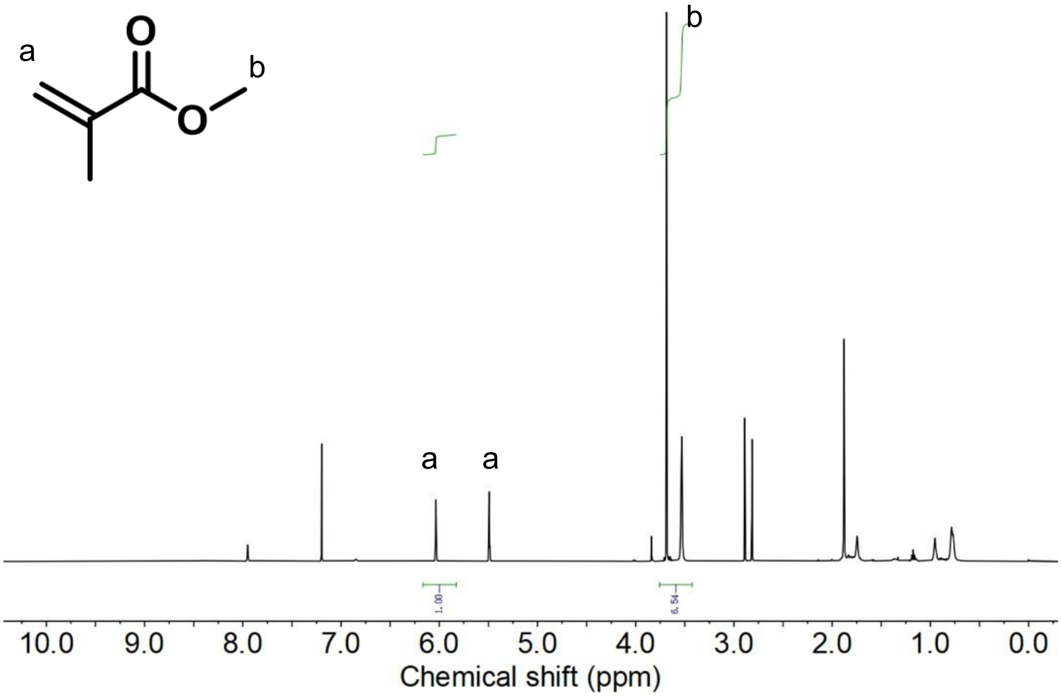

Supplementary Fig. 21 ^{1}H NMR spectroscopy in $CDCl_3$ of MMA monomer after Process 2

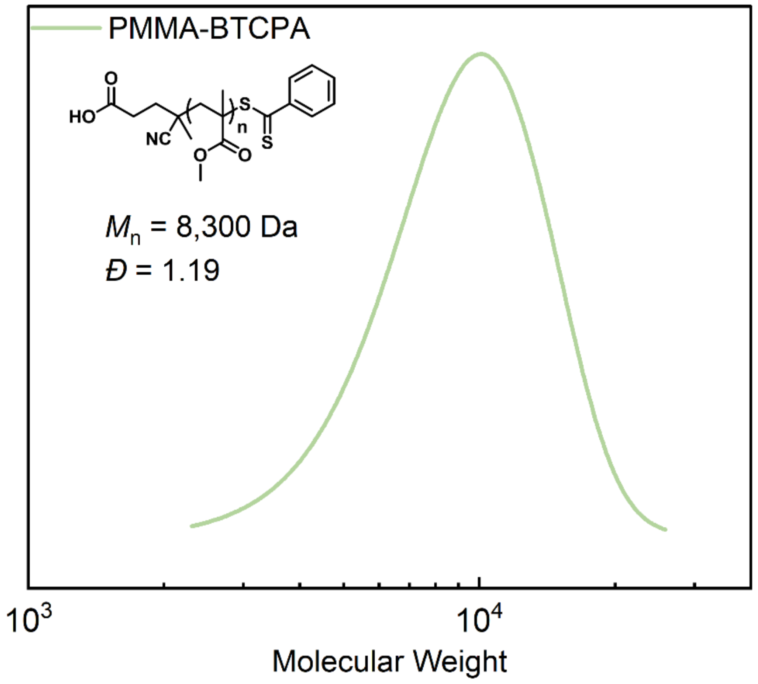

Supplementary Fig. 22 GPC trace of MMA monomers after Process 2

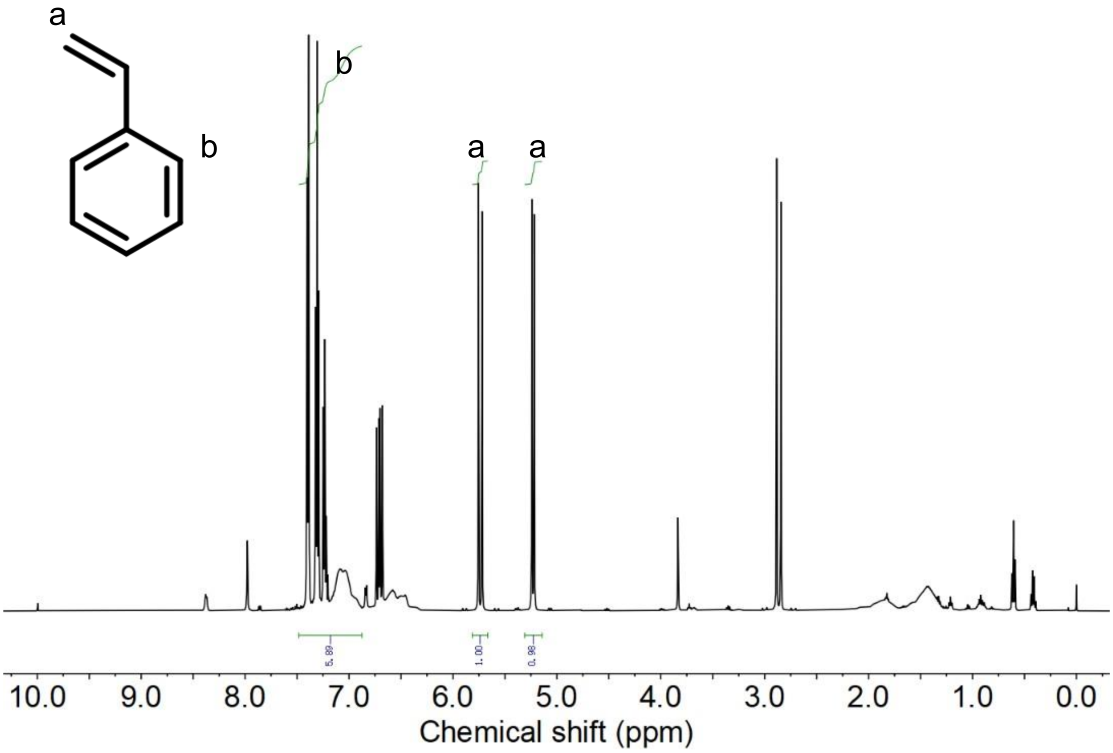

Supplementary Fig. 23 ^{1}H NMR spectroscopy in $CDCl_3$ of styrene monomer after Process 2

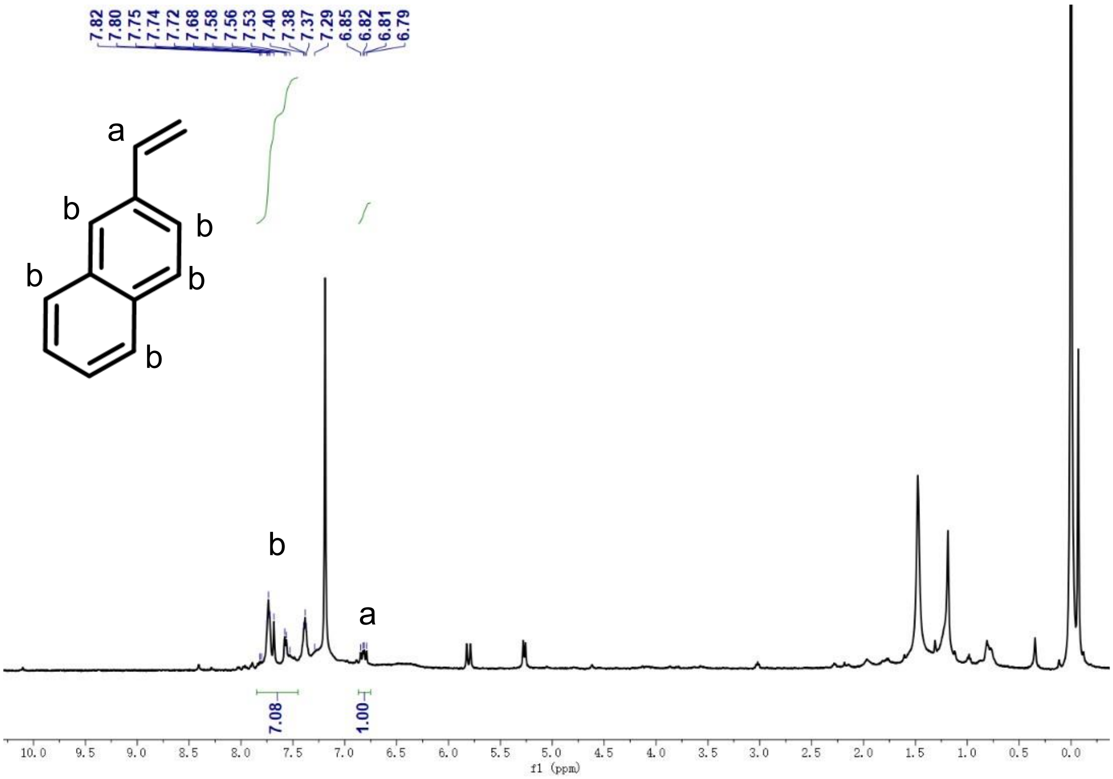

Supplementary Fig. 24 ^{1}H NMR spectroscopy in $CDCl_3$ of vinyl naphthalene monomer after Process 2

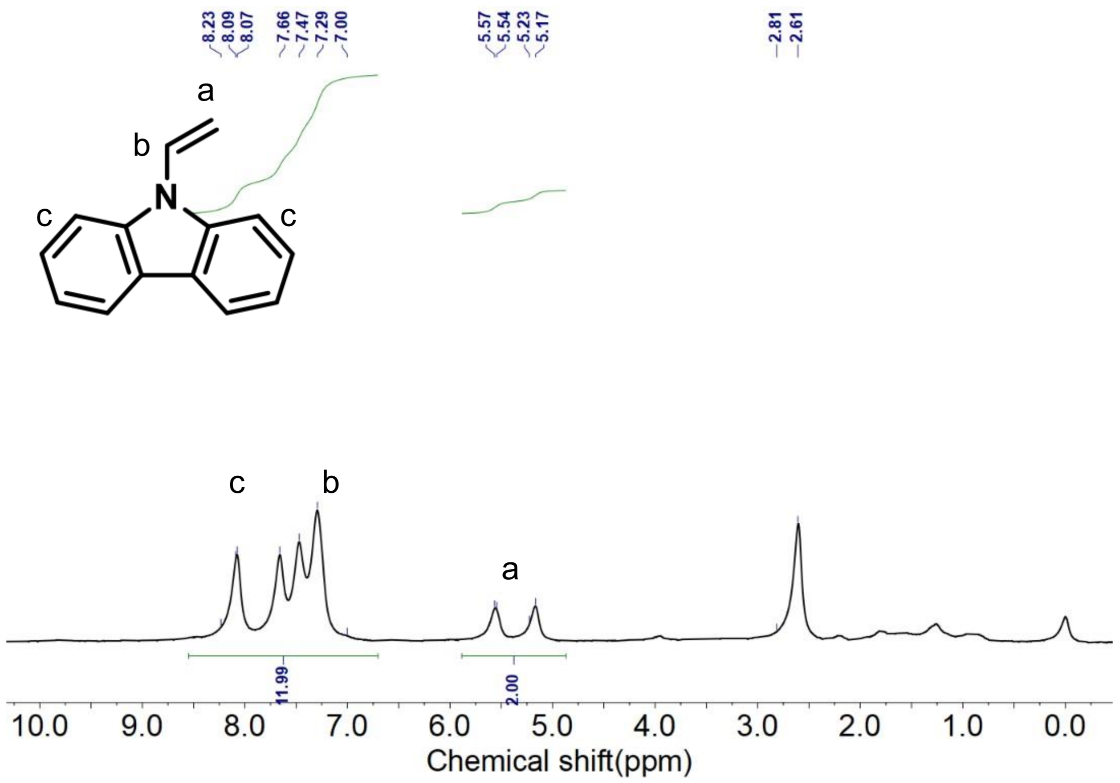

Supplementary Fig. 25 ^{1}H NMR spectroscopy in $CDCl_3$ of vinyl carbazole monomer after Process 2

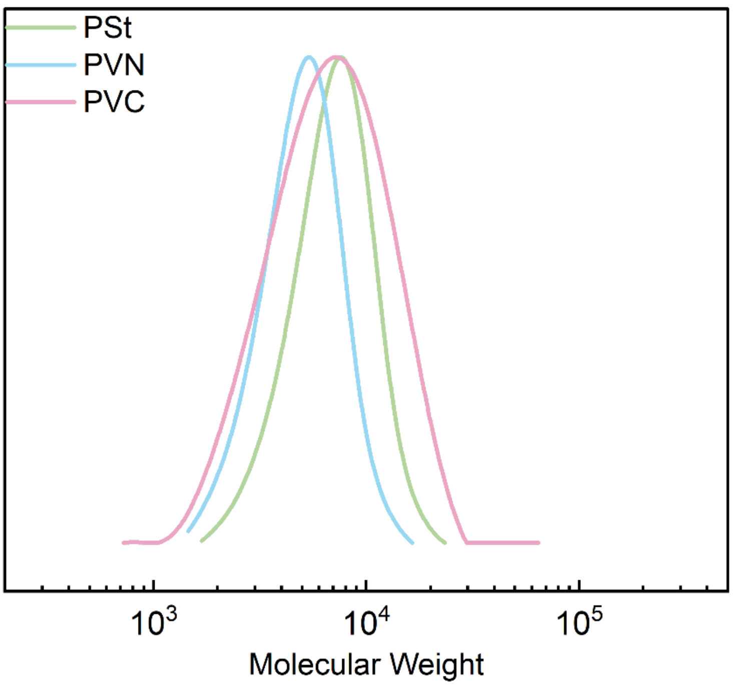

Supplementary Fig. 26 GPC traces of styrene, vinyl naphthalene and vinyl carbazole after Process 2

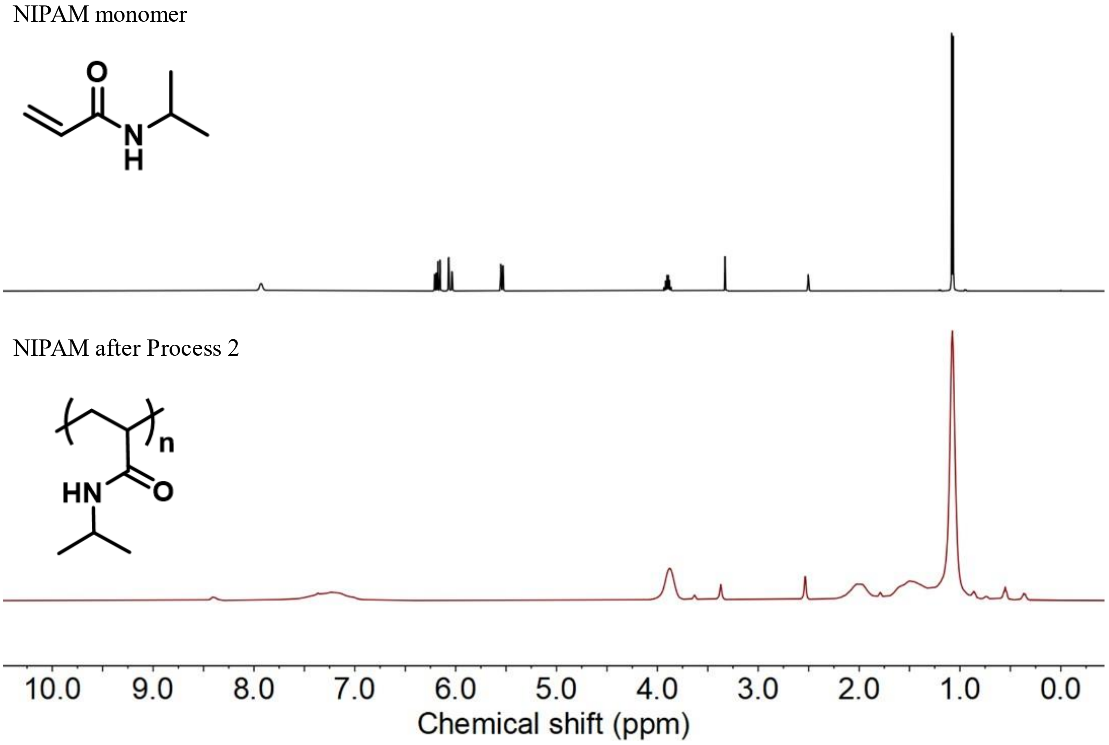

Supplementary Fig. 27 ^{1}H NMR spectra in DMSO-d6 of NIPAM monomer after Process 3

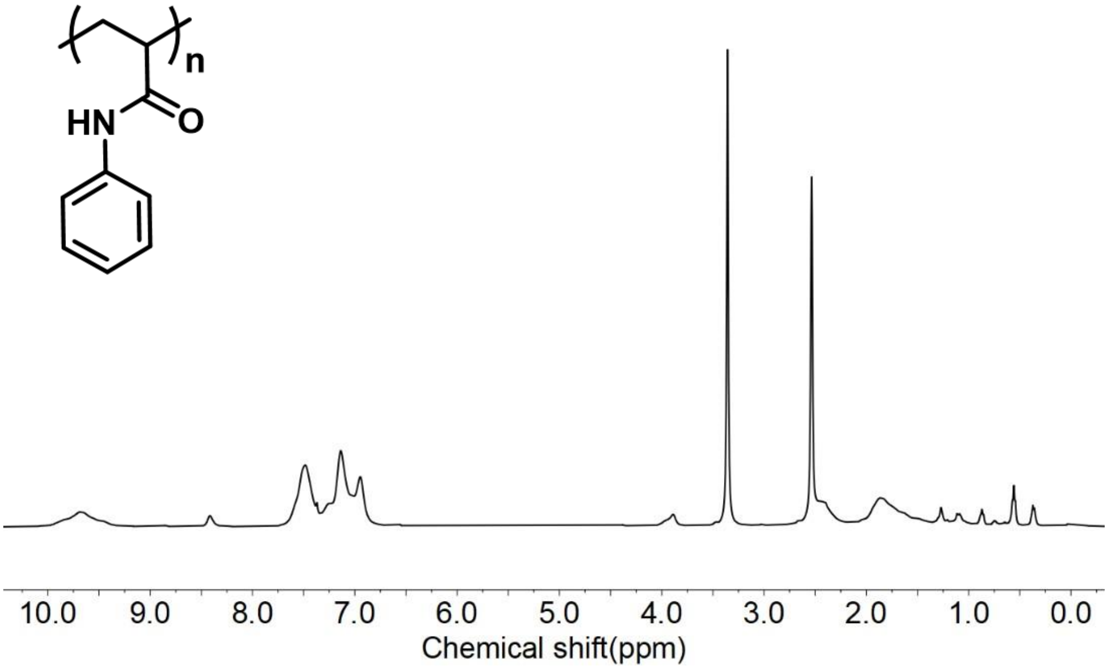

Supplementary Fig. 28 ^{1}H NMR spectroscopy in DMSO-d6 of NPA monomer after Process 3

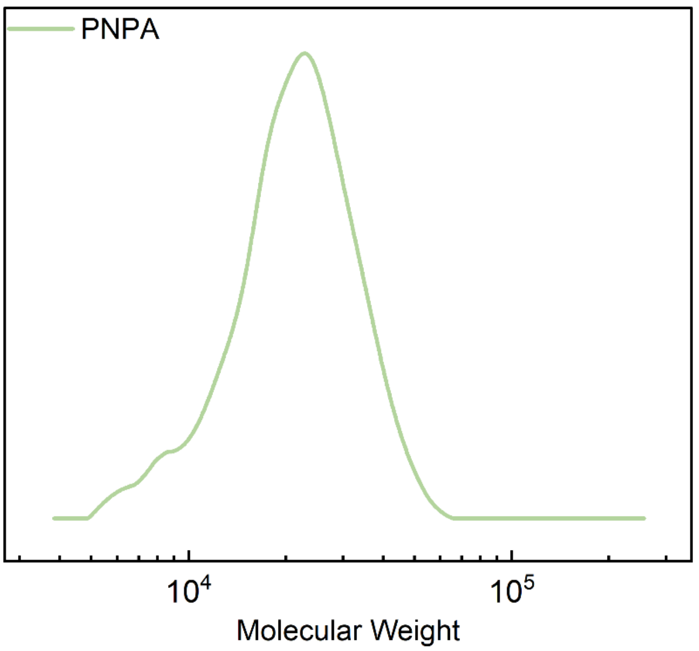

Supplementary Fig. 29 GPC trace (in DMF solution) of NPA monomer after Process 3

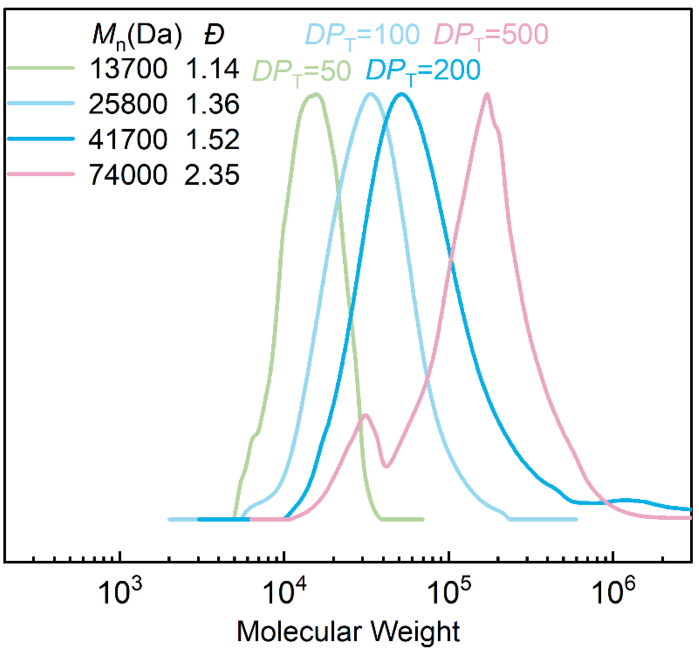

Supplementary Fig. 30 GPC traces (in DMF solution) of NIPAM with different DP_T after Process 3

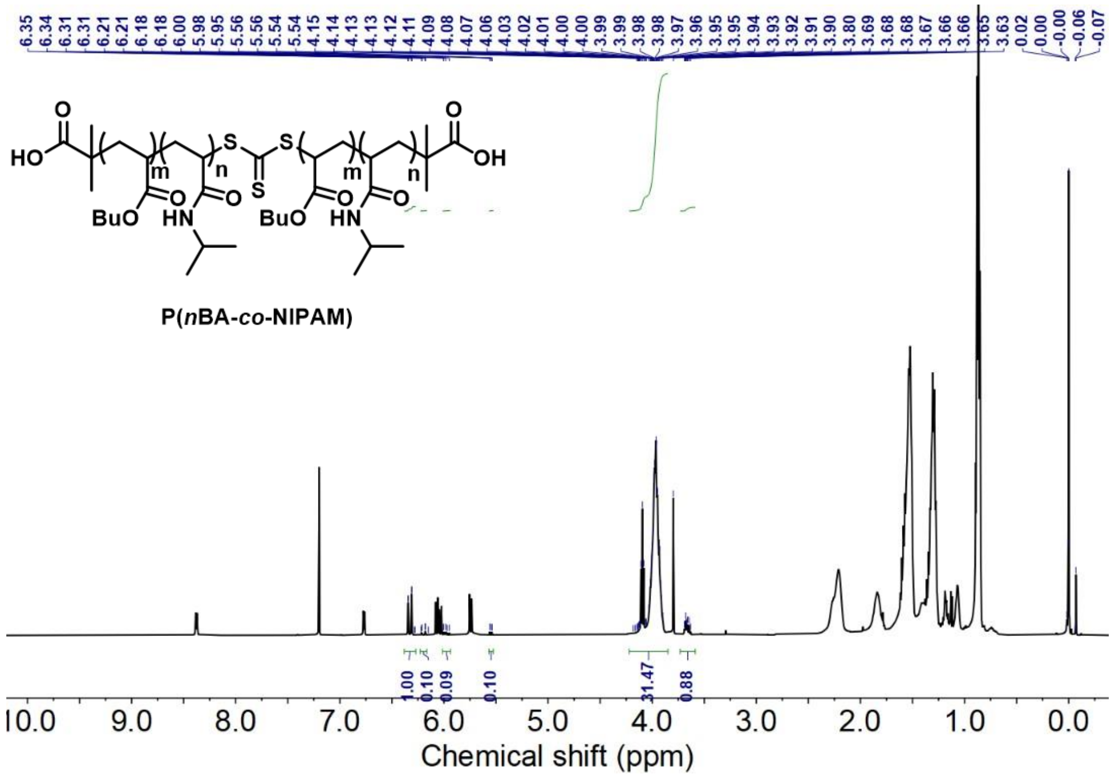

Supplementary Fig. 31 ^{1}H NMR spectroscopy in $CDCl_3$ of the copolymerization of *n*BA and NIPAM based on aerobic mechano-RAFT

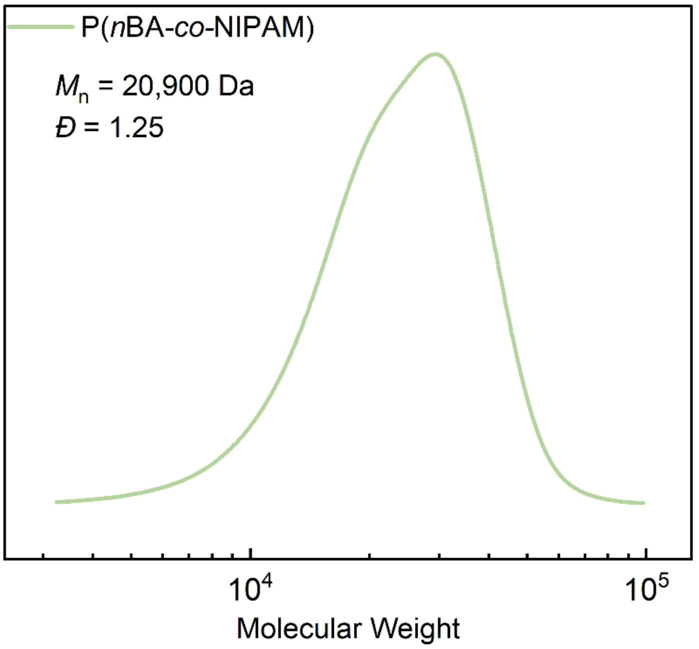

Supplementary Fig. 32 GPC trace of the copolymerization of *n*BA and NIPAM based on aerobic mechano-RAFT

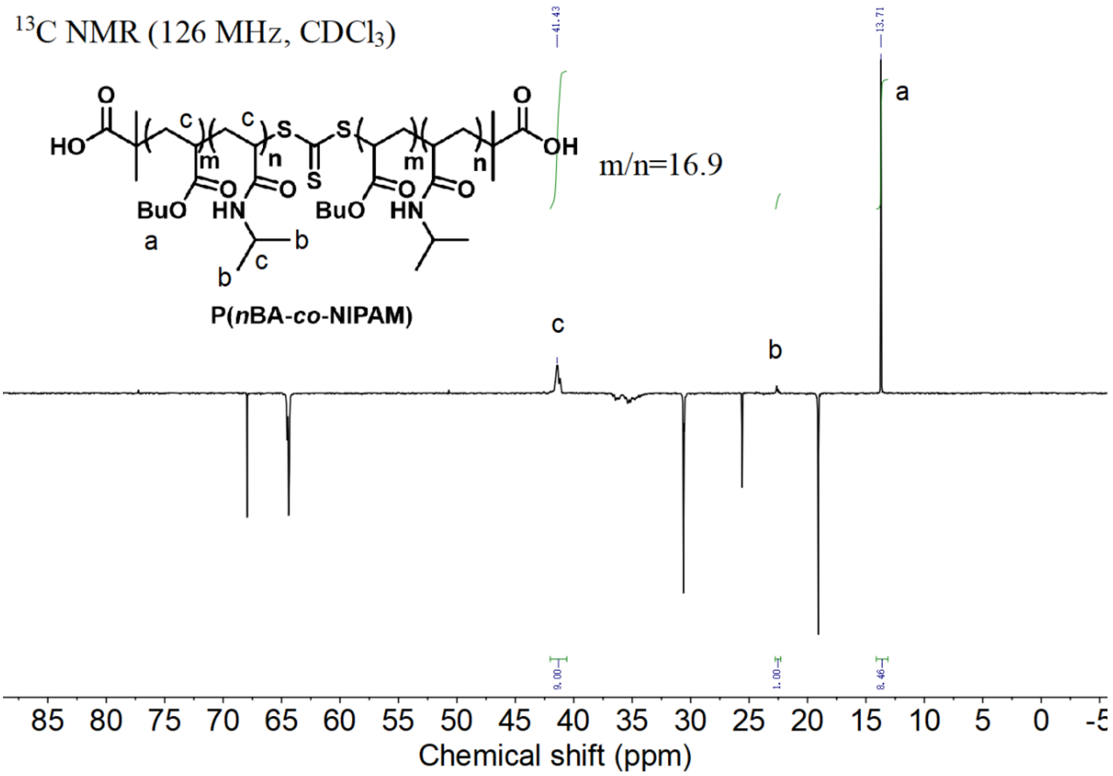

Supplementary Fig. 33 ^{13}C NMR spectroscopy (DEPT135, CH_3/CH positive and CH_2 negative, in $CDCl_3$) of the copolymerization of *n*BA and NIPAM based on aerobic mechano-RAFT

Supplementary Table 1 Aerobic mechano-RAFT with different initiator dosages

Entry[a]	Monomer	[CTA]:[I]	Conversion[b]	$M_{n,th}$ (Da)	$M_{n,GPC}$ (Da)	$Đ$[c]
1	*n*BA	1:1	<11%	/	/	/
2	*n*BA	1:3	30%	7900	18900	1.08
3	*n*BA	1:5	69%	17900	22400	1.10
4	*n*BA	1:6	51%	13300	19400	1.09

[a] Reaction conditions: [M]:[BTPA]:[Et_3B-PyOMe]=200:1:X, no LAG, ball milling (35 mL zirconium oxide jar, 8 mm zirconium oxide grinding ball, 30 Hz), Reaction time-2 h. [b] Conversion was determined by ^{1}H NMR spectroscopy. [c] M_n and $Đ$ were determined by GPC.

Supplementary Table 2 Aerobic mechano-RAFT with different LAG

Entry[a]	Monomer	LAG	Conversion[b]	$M_{n,th}$ (Da)	$M_{n,GPC}$ (Da)	$Đ$[c]
1	*n*BA	NO	69%	17900	22400	1.10
2	*n*BA	DMF	81%	21000	20800	1.11
3	*n*BA	DMSO	76%	19700	4760	3.08
4	*n*BA	Acetone	77%	20000	21500	1.12

5	*n*BA	Dioxane	81%	21000	23000	1.12
6	*n*BA	MeCN	76%	19700	21800	1.09

[a] Reaction conditions: [M]:[BTPA]:[Et_3B-PyOMe]=200:1:5, 100 μL (0.03 mL/g) as LAG, ball milling (35 mL zirconium oxide jar, 8 mm zirconium oxide grinding ball, 30 Hz), Reaction time-2 h. [b] Conversion was determined by ^{1}H NMR spectroscopy. [c] M_n and $Đ$ were determined by GPC.

Supplementary Table 3 Aerobic mechano-RAFT with different CTAs

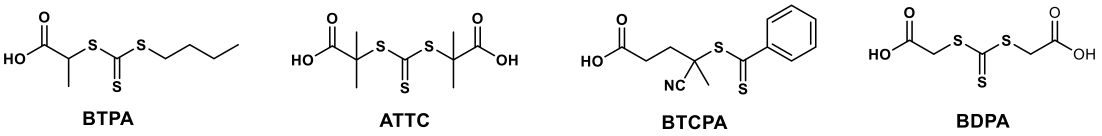

Entry[a]	Monomer	**CTA**	Conversion[b]	$M_{n,th}$ (Da)	$M_{n,GPC}$ (Da)	$Đ$[c]
1	*n*BA	BTPA	81%	21000	20800	1.11
2	*n*BA	ATTC	89%	23000	18100	1.16
3	*n*BA	BTCPA	13%	3600	1900	2.27
4	*n*BA	BDPA	92%	23800	89000	1.79

[a] Reaction conditions: [M]:[CTA]:[Et_3B-PyOMe]=200:1:5, 100 μL (0.03 mL/g) DMF as LAG, ball millling (35 mL zirconium oxide jar, 8 mm zirconium oxide grinding ball, 30 Hz), Reaction time-2 h. [b] Conversion was determined by ^{1}H NMR spectroscopy. [c] M_n and $Đ$ were determined by GPC.

Supplementary Table 4 Aerobic mechano-RAFT with different milling balls

Entry[a]	Monomer	Milling ball	Conversion[b]	$M_{n,th}$ (Da)	$M_{n,GPC}$ (Da)	$Đ$[c]
1	*n*BA	4*8mm	48%	12500	16500	1.10
2	*n*BA	6*8mm	53%	13800	20100	1.11
3	*n*BA	4*10mm	52%	13600	21000	1.09

[a] Reaction conditions: [M]:[BTPA]:[Et_3B-PyOMe]=200:1:5, 100 μL (0.03 mL/g) DMF as LAG, ball millling (35 mL zirconium oxide jar, zirconium oxide grinding ball, 30 Hz), Reaction time-2 h. [b] Conversion was determined by ^{1}H NMR spectroscopy. [c] M_n and $Đ$ were determined by GPC.

Supplementary Table 5 Aerobic mechano-RAFT with different ambient temperature

Entry[a]	Monomer	Temperature/°C	Conversion[b]	$M_{n,th}$ (Da)	$M_{n,GPC}$ (Da)	$Đ$[c]
1	*n*BA	20	65%	16900	16900	1.09
2	*n*BA	25	65%	16900	20800	1.11
3	*n*BA	30	89%	23000	29900	1.09

[a] Reaction conditions: [M]:[BTPA]:[Et_3B-PyOMe]=200:1:5, 100 μL (0.03 mL/g) DMF as LAG, ball millling (35 mL zirconium oxide jar, zirconium oxide grinding ball, 30 Hz), Reaction time-2 h. [b] Conversion was determined by ^{1}H NMR spectroscopy. [c] M_n and $Đ$ were determined by GPC.

Supplementary Table 6 Aerobic mechano-RAFT with different air volume

Entry[a]	Monomer	Jar	Conversion[b]	$M_{n,th}$ (Da)	$M_{n,GPC}$ (Da)	$Đ$[c]
1	*n*BA	35 ml	81%	21000	20800	1.11
2	*n*BA	25 ml	81%	21000	20500	1.11

[a] Reaction conditions: [M]:[BTPA]:[Et_3B-PyOMe]=200:1:5, 100 μL (0.03 mL/g) as LAG, ball millling (X mL zirconium oxide jar, 8 mm zirconium oxide grinding ball, 30 Hz), Reaction time-2 h. [b] Conversion was determined by ^{1}H NMR spectroscopy. [c] M_n and $Đ$ were determined by GPC.

Synthesis methods

Materials

Triethylborane (1.0 M solution in THF, Safeseal) and 4-Methoxypyridine (RG,>98%) were purchased from Adamas. *N*, *N*-dimethylformamide (DMF), dimethyl sulfoxide (DMSO), acetonitrile (MeCN), dichloromethane (DCM), acetone, and dioxane were purchased from Energy Chemical and dried before use. Other chemicals were purchased from commercial sources and used as received unless stated otherwise. All the monomers were passed through a column of basic alumina (Fisher Scientific) prior to use to remove inhibitor. Ultrapure Water was generated from a Milli-Q Integral Water Purification System.

Synthesis Details

Synthesis of Triethylborane/4-methoxypyridine complex (Et_3B-PyOMe).

4-methoxypyridine complex (2.18 g ,20 mmol) was placed in a 50 mL round bottom flask filled with dry argon, and then triethylborane/THF solution (20 mL) was completely dropwise added to a stirred solution of 4-methoxypyridine cooled in an ice-water bath. The mixture was stirred for 30 min followed by solvent removal by a rotary evaporation. The yellow liquid product was obtained (4.11 g, 99.3% yield). ^{1}H NMR (500 MHz, Chloroform-*d*) δ 8.30 – 8.25 (m, 2H), 6.90 – 6.81 (m, 2H), 3.86 (s, 3H), 0.51 (t, J = 7.6 Hz, 9H), 0.32 (q, J = 7.8 Hz, 6H).

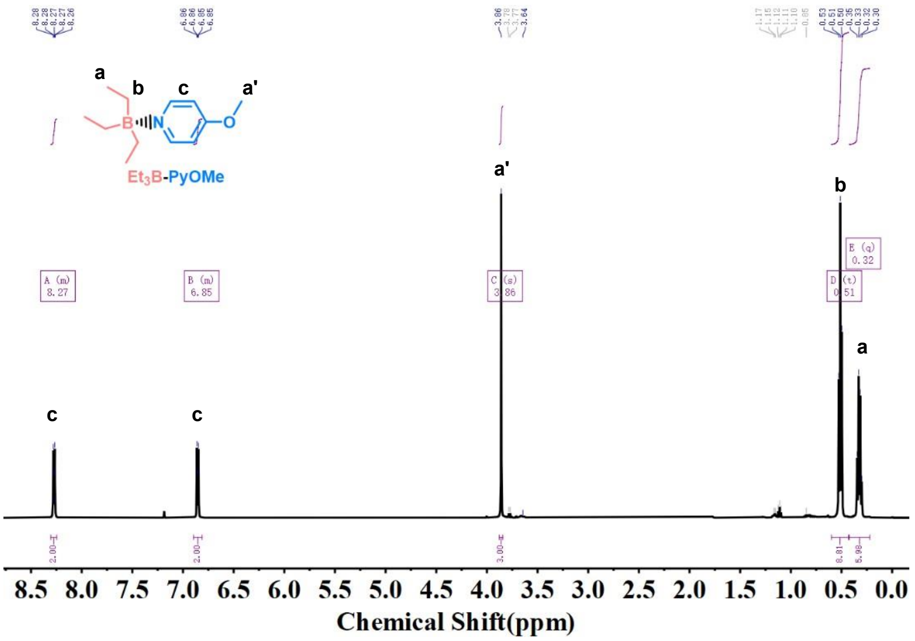

The preparation process of BTPA chain transfer agent.

According to the reported literature[1], 200 mol of sodium hydroxide/water solution (50 wt.%) was added dropwise to butanethiol/water solution (200 mmol butanethiol) in a 250 ml round bottom flask. Then 10ml of acetone and 225 mM of carbon disulfide was added respectively to the reaction and stirred for 30 min. 205 mM of 2-bromopropionic acid and 16.4 ml of 50 % aqueous sodium hydroxide solution was added drop by drop in an ice bath and reacted for 24 hours at room temperature. Add 60 ml of 10 M HCl drop by drop in an ice bath until a yellow oily substance is separated. The yellow crude product was separated out and washed with cold water and dried. Afterwards the crude product was recrystallized in n-hexane to get a yellow crystalline product. ^{1}H NMR (500 MHz, Chloroform-*d*) δ 4.87 (q, *J* = 7.4 Hz, 1H), 3.38 (t, *J* = 7.4 Hz, 2H), 1.75 – 1.58 (m, 5H), 1.43 (dt, *J* = 14.8, 7.4 Hz, 2H), 0.94 (t, *J* = 7.4 Hz, 3H).

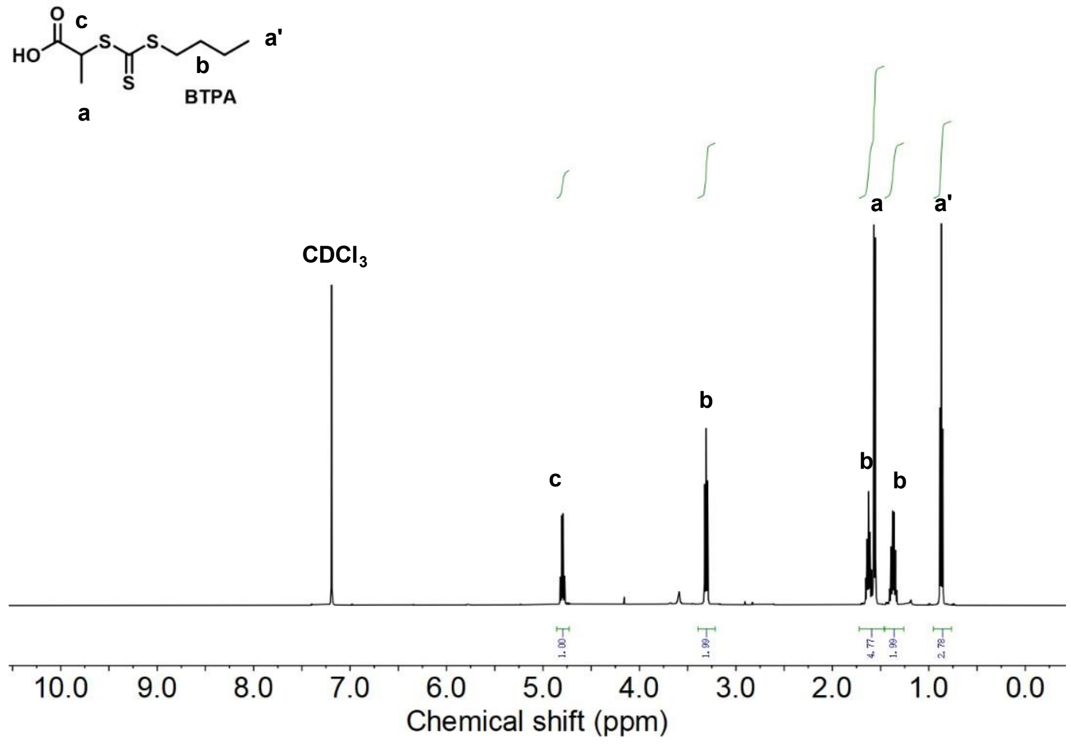

The preparation process of ATTC chain transfer agent.

Synthesis of ATTC was followed according to references[2]. 99 mmol of acetone, 9 mmol of trichloromethane and 13 ml of hexane were mixed in a 250 ml round bottom flask cooling in an ice bath. 0.45 mmol of n-tetrabutylammonium sulphate was added and stirred for 30 minutes. After reaction, 40 mmol carbon disulphide was added dropwise. And then 50 ml of 44% aqueous sodium hydroxide solution was added to the reaction solution within 30 minutes under below 15°C. After overnight reaction, the precipitate was dissolved by adding deionized water, concentrated HCl

was added to adjust pH below 4 under ice bath and reacted for 15 min. The crude product was filtered, washed and dried, then recrystallized from ethanol to get a yellow solid product. ^{1}H NMR (500 MHz, Chloroform-*d*) δ 1.70 (s, 12H).

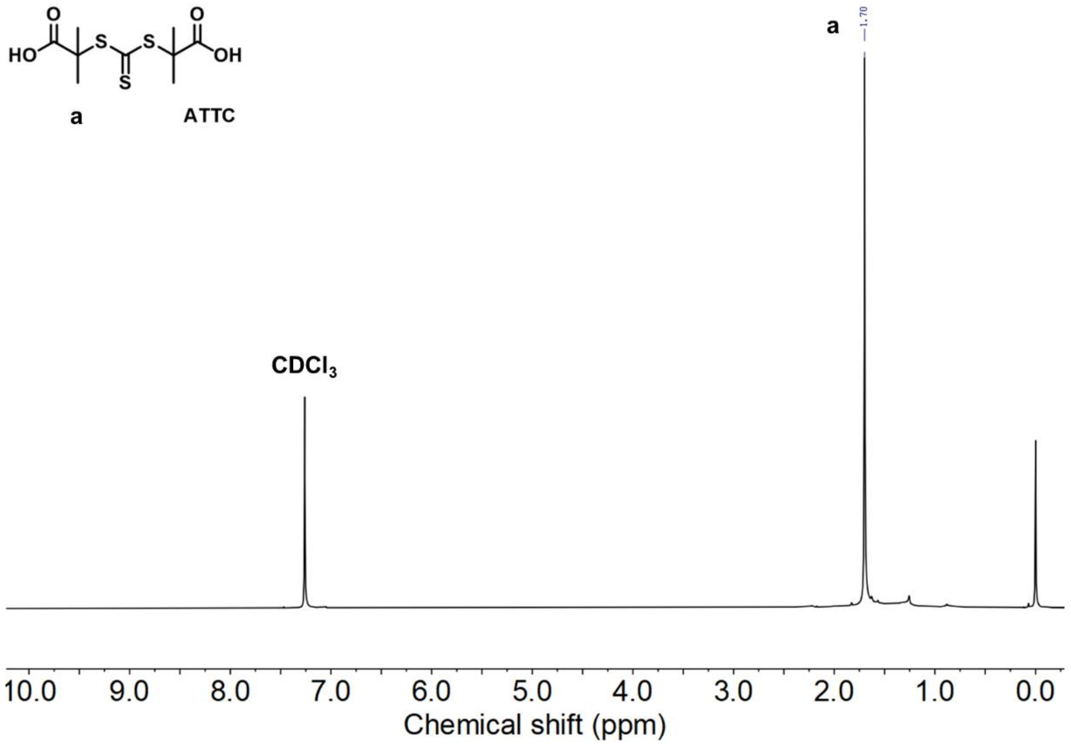

Supplementary references

1 Shanmugam, S., Cuthbert, J., Kowalewski, T., Boyer, C. & Matyjaszewski, K. Catalyst-Free Selective Photoactivation of RAFT Polymerization: A Facile Route for Preparation of Comblike and Bottlebrush Polymers. *Macromolecules* **51**, 7776-7784 (2018).

2 Chakma, P. *et al.* Mechanoredox Catalysis Enables a Sustainable and Versatile Reversible Addition-Fragmentation Chain Transfer Polymerization Process. *Angewandte Chemie International Edition* **62**, e202215733 (2023).
